# Lactoferrin as Antiviral Treatment in COVID-19 Management: Preliminary Evidence

**DOI:** 10.3390/ijerph182010985

**Published:** 2021-10-19

**Authors:** Elena Campione, Caterina Lanna, Terenzio Cosio, Luigi Rosa, Maria Pia Conte, Federico Iacovelli, Alice Romeo, Mattia Falconi, Claudia Del Vecchio, Elisa Franchin, Maria Stella Lia, Marilena Minieri, Carlo Chiaramonte, Marco Ciotti, Marzia Nuccetelli, Alessandro Terrinoni, Ilaria Iannuzzi, Luca Coppeta, Andrea Magrini, Sergio Bernardini, Stefano Sabatini, Felice Rosapepe, Pier Luigi Bartoletti, Nicola Moricca, Andrea Di Lorenzo, Massimo Andreoni, Loredana Sarmati, Alessandro Miani, Prisco Piscitelli, Ettore Squillaci, Piera Valenti, Luca Bianchi

**Affiliations:** 1 Dermatology Unit, Department of Systems Medicine, Tor Vergata University Hospital, 00133 Rome, Italy; caterinalanna.cl@gmail.com (C.L.); terenziocosio@gmail.com (T.C.); luca.bianchi@uniroma2.it (L.B.); 2 Department of Public Health and Infectious Diseases, University of Rome “La Sapienza”, 00185 Rome, Italy; luigi.rosa@uniroma1.it (L.R.); mariapia.conte@uniroma1.it (M.P.C.); piera.valenti@uniroma1.it (P.V.); 3 Structural Bioinformatics Group, Department of Biology, University of Rome “Tor Vergata”, 00133 Rome, Italy; federico.iacovelli@uniroma2.it (F.I.); alice.romeo@uniroma2.it (A.R.); falconi@uniroma2.it (M.F.); 4 Department of Molecular Medicine, University of Padova, 35122 Padova, Italy; claudia.delvecchio@unipd.it (C.D.V.); elisa.franchin@unipd.it (E.F.); 5 Department of Experimental Medicine, Tor Vergata University Hospital, 00133 Rome, Italy; lia.mariastella@gmail.com (M.S.L.); minieri@med.uniroma2.it (M.M.); alessandro.terrinoni@uniroma2.it (A.T.); 6 Department of Statistics, University of Rome Tor Vergata, 00133 Rome, Italy; chiaramonte.carlo43@gmail.com; 7 Virology Unit, Tor Vergata University Hospital, 00133 Rome, Italy; marco.ciotti@ptvonline.it; 8 Laboratory Medicine, Department of Experimental Medicine and Surgery, Tor Vergata University Hospital, 00133 Rome, Italy; marzianuccetelli@yahoo.com (M.N.); bernardini@med.uniroma2.it (S.B.); 9 Occupational Medicine Department, University of Rome “Tor Vergata”, 00133 Rome, Italy; ilariaiannuzzi@gmail.com (I.I.); luca.coppeta@gmail.com (L.C.); andrea.magrini@uniroma2.it (A.M.); 10 Villa dei Pini Hospital, 00042 Anzio, Italy; stefanosabatini@gmail.com (S.S.); nicolamoricca@gmail.com (N.M.); 11 Pineta Grande Hospital, 81030 Caserta, Italy; frosapepe@gmail.com; 12 Fimmg Provincial, 00144 Rome, Italy; pl.bartoletti@gmail.com; 13 Infectious Disease Unit, Tor Vergata University Hospital, 00133 Rome, Italy; andrea.dilorenzo@alumni.uniroma2.eu (A.D.L.); andreoni@uniroma2.it (M.A.); sarmati@med.uniroma2.it (L.S.); 14 Department of Environmental Sciences and Policy, University of Milan, 20133 Milan, Italy; alessandro.miani@unimi.it; 15 UNESCO Chair on Health Education and Sustainable Development, University of Naples Federico II, 80131 Naples, Italy; piscitelli@unescochairnapoli.it; 16 Department of Diagnostic and Molecular Imaging, Radiation Therapy and Interventional Radiology, University Hospital Tor Vergata, 00133 Rome, Italy; ettore.squillaci@uniroma2.it

**Keywords:** bovine lactoferrin, liposomal bovine lactoferrin, COVID-19, SARS-CoV-2, IL-6, ferritin, D-dimers

## Abstract

Lactoferrin (Lf), a multifunctional cationic glycoprotein synthesized by exocrine glands and neutrophils, possesses an in vitro antiviral activity against SARS-CoV-2. Thus, we conducted an in vivo preliminary study to investigate the antiviral effect of oral and intranasal liposomal bovine Lf (bLf) in asymptomatic and mild-to-moderate COVID-19 patients. From April 2020 to June 2020, a total of 92 mild-to-moderate (67/92) and asymptomatic (25/92) COVID-19 patients were recruited and divided into three groups. Thirty-two patients (14 hospitalized and 18 in home-based isolation) received only oral and intranasal liposomal bLf; 32 hospitalized patients were treated only with standard of care (SOC) treatment; and 28, in home-based isolation, did not take any medication. Furthermore, 32 COVID-19 negative, untreated, healthy subjects were added for ancillary analysis. Liposomal bLf-treated COVID-19 patients obtained an earlier and significant (*p* < 0.0001) SARS-CoV-2 RNA negative conversion compared to the SOC-treated and untreated COVID-19 patients (14.25 vs. 27.13 vs. 32.61 days, respectively). Liposomal bLf-treated COVID-19 patients showed fast clinical symptoms recovery compared to the SOC-treated COVID-19 patients. In bLf-treated patients, a significant decrease in serum ferritin, IL-6, and D-dimers levels was observed. No adverse events were reported. These observations led us to speculate a potential role of bLf in the management of mild-to-moderate and asymptomatic COVID-19 patients.

## 1. Introduction

Severe acute respiratory syndrome coronavirus 2 (SARS-CoV-2), possessing more than 80% identity to SARS-CoV [1,2], is a highly transmissible pathogen that causes the acute respiratory disease named ‘coronavirus disease 2019’ (COVID-19).

SARS-CoV-2 is a spherical, enveloped virus possessing a single-stranded, positive-sense RNA genome with four fundamental structural proteins, such as spike glycoprotein, small envelope protein, matrix protein, and nucleocapsid protein [3]. The homotrimeric spike glycoprotein is located on the envelope and comprises two subunits (S1 and S2) in each spike monomer [4]; it is involved in the binding to the cell receptor angiotensin-converting enzyme 2 (ACE2), through its S1 subunit, and in membrane fusion, through the S2 subunit [1,5]. Apart from ACE2, the heparan sulfate proteoglycans (HSPGs) located on the cell surface have also been recognized as binding sites for SARS-CoV [6] and are pivotal also for SARS-CoV-2 in the early attachment phase [7]. In humans, high ACE2 expression is found in lungs and several other tissues [8,9,10], thus explaining the severity and ubiquity of SARS-CoV-2 infection [11].

The main symptoms of COVID-19 are fever (83–98%), cough (50–82%), fatigue (25–44%), shortness of breath (19–55%), and muscle soreness (11–44%) [12,13]. Some patients may also manifest sore throat, nausea, vomiting, diarrhea, headache, ageusia, and anosmia a few days before the occurrence of fever, suggesting that fever is critical but not the only initial symptom of infection [12]. In contrast, many patients only have a mild fever, mild fatigue, or even no symptoms [13,14,15,16]. About 80% of COVID-19 patients manifest a mild respiratory illness and could be treated by outpatient care, while about 15% of patients need hospitalization for moderate to severe pneumonia, requiring admission to an intensive care unit (ICU), especially the patients suffering from comorbidities such as cardiovascular disease, hypertension, diabetes, and renal disease [17].

COVID-19 also induces massive release of cytokines (cytokine storm) [18], increased coagulation state [19,20], hemoglobin damage [21], and dysregulation of iron homeostasis [15], including iron overload [22], which is an important factor in the pathogenesis of viral infections [23].

At the beginning of the pandemic, no drug was proven to be safe and effective for treating COVID-19 [24]. However, several standard of care (SOC) regimens had been proposed for the management of the pandemic. Lopinavir/ritonavir has been proposed as a treatment for COVID-19 on the basis of in vitro activity, preclinical studies, and observational studies [25].

Lopinavir/ritonavir is an inhibitor of SARS-CoV 3CLpro in vitro, and this protease appears highly conserved in SARS-CoV-2 [26,27].

Hydroxychloroquine and chloroquine are two other drugs which were used during the early phases of the pandemic under the United States Food and Drug Administration (US FDA) Emergency Use Authorizations, on the basis of their ability to inhibit fusion of SARS-CoV-2 and the host cell membranes [28]. Moreover, chloroquine inhibits glycosylation of the cellular ACE2 receptor, which may interfere with binding of SARS-CoV to the cell receptor [29].

Recently, lactoferrin (Lf) is emerging as a pleiotropic glycoprotein, belonging to innate immunity, able to simultaneously counteract the inflammatory and iron homeostasis disorders caused by bacterial and viral attacks [30].

Lf, synthesized by exocrine glands and neutrophils, is present in human milk and secretions [30,31]. Human Lf (hLf) has a high homology of sequence with bovine Lf (bLf), which, in turn, possesses identical functions with hLf. For this reason, bLf, available in large quantities, is used in all in vitro and in vivo studies, having been approved as a Generally Recognized as Safe (GRAS) compound by the US FDA [32] and as a dietary supplement by the European Food Safety Authority [33].

Indeed, three promising in vitro studies on SARS-CoV-2 have proved that bLf inhibits the early phase of viral infection [7,34,35].

BLf has four pleiotropic activities: chelation of two ferric ions per molecule, interaction with anionic compounds, entry inside the nucleus, and modulation of iron and inflammatory homeostasis [30]. The ability of bLf to chelate two ferric ions per molecule influences bacterial and viral replication and hinders reactive oxygen species formation [31,36,37]. The binding of bLf to anionic surface compounds, due to its cationic feature, is associated with host protection against bacterial and viral adhesion and entry [31,36]. Moreover, the entrance of bLf into host cells and its translocation into the nucleus [38,39] are related to its anti-inflammatory function [40,41,42]. Furthermore, bLf ability to restore iron homeostasis, perturbed by viral infection and inflammation [23], is associated with its ability to chelate iron, decrease iron overload, diminish interleukin (IL)-6 levels, and modulate the expression of iron proteins. As a matter of fact, iron homeostasis is maintained by the expression of some iron proteins, such as transferrin, ferroportin, hepcidin, and ferritin. The disorders of iron homeostasis, induced by infection and inflammation, increase intracellular iron concentration, thus favoring viral replication [23,43]. Moreover, hLf seems to modulate plasminogen activation and control the coagulation cascade with a remarkable antithrombotic activity [44], a very frequent complication of SARS-CoV-2 infection [45].

Here, we present the results of a preliminary clinical trial on the efficacy of an oral and intranasal liposomal bLf formulation in asymptomatic and mild-to-moderate COVID-19 patients.

## 2. Materials and Methods

### 2.1. Study Population

A clinical trial was conducted to investigate the effect and tolerability of an oral and intranasal liposomal bLf formulation as treatment in asymptomatic and mild-to-moderate COVID-19 patients. From April 2020 to June 2020, a total of 92 patients with confirmed COVID-19 infection at reverse transcriptase real time (rRT)-PCR naso-oropharyngeal swab were recruited to participate in this preliminary study. Patients were divided into three groups: 32 patients, 14 hospitalized and 18 in home-based isolation, received oral and intranasal liposomal bLf; 32 hospitalized patients were treated only with a SOC regimen (hydroxychloroquine and lopinavir/darunavir); while 28 patients in home-based isolation did not receive any anti-COVID-19 treatment. Hospitalized patients were treated with azithromycin before admission to hospital by general practitioners.

All recruited patients were asymptomatic or presented mild-to-moderate symptoms. A mild-to-moderate disease was defined by less severe clinical symptoms with no evidence of pneumonia and not requiring ICU [46].

A control group of 32 healthy volunteers, with negative COVID-19 infection at rRT-PCR naso-oropharyngeal swab, was involved in this preliminary study for ancillary analysis.

### 2.2. Enrollment Criteria

Eligible patients were over 20 years old, with a confirmed COVID-19 rRT-PCR from naso-oropharyngeal swab and blood oxygen saturation (SpO_2_) > 93% or Horowitz index (PaO_2_/FiO_2_) > 300 mmHg. Patients had not been treated previously for SARS-CoV-2. Exclusion criteria included pregnancy and breastfeeding, nitric oxide and nitrates assumptions, known allergy to milk proteins, a medical history of bronchial hyperactivity, or pre-existing respiratory diseases. ICU COVID-19 in-patients were excluded.

Eligibility for the SOC regimen in hospitalized patients was verified at admission by electrocardiogram (ECG) due to the risk of QT interval prolongation and torsade de pointes associated with this drug regimen [47,48,49].

During the recovery, all SOC-treated patients were monitored through peripheral oxygen saturation, ECG, and blood pressure by telemetry.

In this preliminary study, performed in the central phase of the pandemic, placebo and liposome unloaded with bLf groups were not considered, following the specific request of our Hospital Ethics Committee.

**Figure 1:**
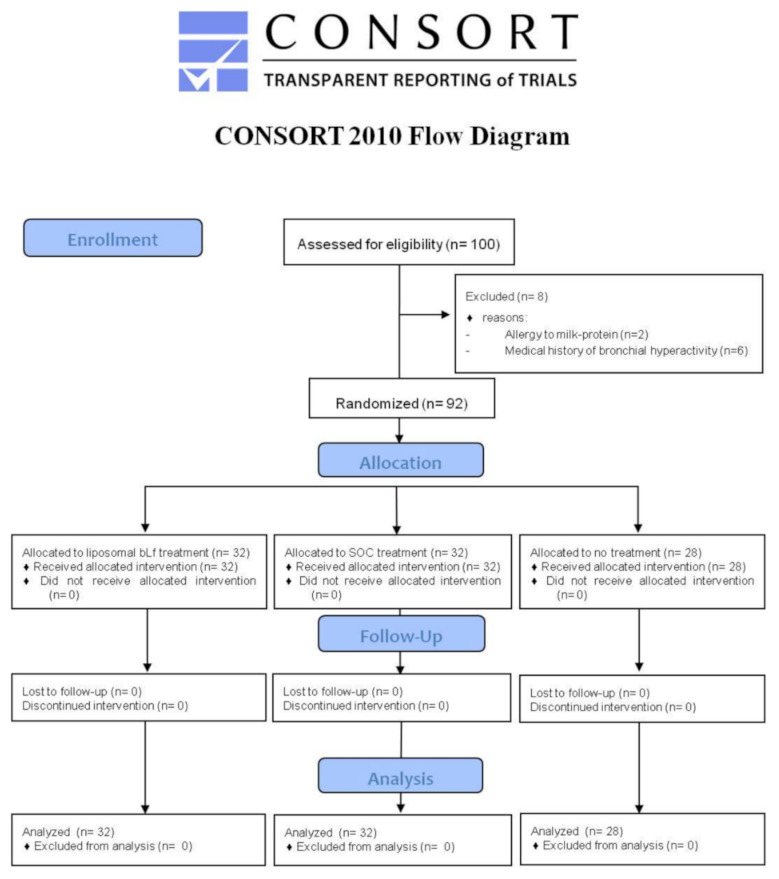
CONSORT diagram of the clinical trial. BLf = bovine lactoferrin; SOC = standard of care.

All patients gave written informed consent after receiving an extensive disclosure of the study purposes and risks. The trial was approved by the Tor Vergata University Hospital Ethics Committee (Code 42/20). It was registered at www.clinicaltrials.gov (Identifier: NCT04475120) (accessed on 2 August 2021) and reported according to CONSORT guidelines (Figure 1).

### 2.3. Patient Groups

Thirty-two patients (14 hospitalized and 18 in home-based isolation) belonging to the first group received oral and intranasal liposomal bLf. BLf capsules for oral use contained 100 mg of bLf encapsulated in liposomes, while bLf nasal spray had about 8 mg/mL of bLf encapsulated in liposomes. BLf, contained in both products, was tested by SDS-PAGE and silver nitrate staining, and its purity was about 95%. The bLf iron saturation was about 5%, as detected via optical spectroscopy at 468 nm based on an extinction coefficient of 0.54 (100% iron saturation, 1% solution). The scheduled dose treatment of liposomal bLf for oral use was 1 g per day for 30 days (10 capsules per day). The liposomal bLf capsules were divided into 3 daily administrations and taken before meals in order to avoid protein degradation due to the low pH of gastric juice during digestion [50]. In addition, the liposomal bLf intranasal formulation was administered 3 times daily (a total of about 16 mg/nostril/day).

Thirty-two hospitalized COVID-19 patients belonging to the second group were only treated with the SOC regimen according to the national guidelines at the time of the enrollment: lopinavir/ritonavir capsules 200/50 mg, 2 × 2/day (alternatively darunavir 800 mg 1 capsule/day + ritonavir 100 mg 1 capsule/day or darunavir/cobicistat 800/150 mg 1 capsule/day) and chloroquine 500 mg, 1 × 2/day or hydroxychloroquine capsule 200 mg, 1 × 2/day. SOC regimens lasted from 5 to 20 days, with timing established according to clinical course.

Twenty-eight COVID-19 patients, in home-based isolation, belonging to the third group did not receive any therapy.

A control group, comprising 32 healthy volunteers, did not receive any treatment or placebo.

Blood samples and clinical assessments were evaluated at baseline (T0) and after 30 days (T2).

### 2.4. Primary Endpoint

The primary endpoint was to assess the mean time length needed to reach a rRT-PCR negative conversion rate of SARS-CoV-2 RNA in each group of COVID-19 patients enrolled in the study.

### 2.5. Secondary Endpoints

The secondary endpoint was to estimate the proportion of patients who achieved disease remission, defined as symptoms recovery.

In addition, safety and tolerability of liposomal bLf for oral and intranasal use were assessed.

### 2.6. Tertiary Endpoint

The tertiary endpoint was an ancillary analysis to evaluate in the three groups the following parameters: the number of red blood cells, leukocytes, neutrophils, monocytes, and platelets; and the serum levels of hemoglobin, ferritin, IL-6, IL-10, tumor necrosis factor (TNF)-α, aspartate aminotransferase (AST), alanine aminotransferase (ALT), adrenomedullin, and D-dimers at T0 and T2 (after 30 days with or without treatment) according to the standard laboratory procedures of the University of Tor Vergata. The values of these parameters were compared with those detected in the healthy volunteers group.

### 2.7. Statistical Analysis

With regard to this preliminary study, descriptive and inferential statistical analyses were performed. The Kolmogorov–Smirnov test was used to test the normal distribution of blood parameters.

The detected parameters at T0 and T2 in the COVID-19 groups (liposomal bLf-, SOC-treated or untreated) were compared with those assayed in the healthy control group using a *t*-test. Data were then analyzed with a significant two-tailed *p*-value ≤ 0.05.

All parameters detected at T0 and T2 in the COVID-19 groups were then compared using a paired *t*-test. In addition, the mean change between T0 and T2 was also assessed using a paired *t*-test. Normally distributed data were then analyzed with a significant *p*-value ≤ 0.05.

## 3. Results

### 3.1. Demographic Data

**Figure 2:**
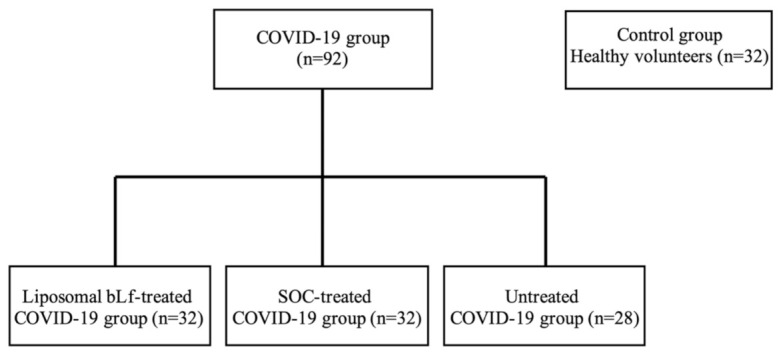
A total of 92 patients with confirmed COVID-19 infection at reverse transcriptase real time (rRT)-PCR were recruited to participate in this study. Patients were divided into three groups according to the administered treatment. Thirty-two patients were treated with liposomal bovine lactoferrin (bLf); another 32 patients received a standard of care (SOC) regimen; while 28 patients did not receive any treatment against COVID-19. As a control group, 32 healthy volunteers with negative COVID-19 infection at rRT-PCR naso-oropharyngeal swab were recruited.

A total of 92 patients with confirmed COVID-19 infection at rRT-PCR naso-oropharyngeal swab were recruited to participate in this preliminary study (Figure 2).

Demographic data of the groups are summarized in Table 1.

Among the 32 liposomal bLf-treated patients, 10 were asymptomatic and 22 had mild-to-moderate symptoms. Among these, 14 patients were hospitalized and 18 were in home-based isolation. The mean age was 54.6 ± 16.9 years old. Fourteen COVID-19 patients were male and 18 female (Table 1). The most prevalent comorbidity was hypertension (28%), followed by cardiovascular diseases (15.6%) and dementia (12.5%).

In the group of 32 hospitalized SOC-treated patients, 3 were asymptomatic and 29 had mild-to-moderate symptoms. The mean age was 49.9 ± 13.2 years old. Seventeen patients were male and 15 female (Table 1). The most prevalent comorbidity was hypertension (15.6%), followed by asthma (12.5%) and hypothyroidism (6.5%).

Among the 28 COVID-19 patients in home-based isolation not taking any drug against COVID-19, 12 patients were asymptomatic and 16 had mild-to-moderate symptoms. All of these patients were in home-based isolation. The mean age was 41.3 ± 11.8 years old. Ten patients were male and 18 female (Table 1). However, four of these patients worsened their clinical profile, with consequent hospitalization.

Among the 32 healthy volunteers (mean age 52.8 ± 15.5 years old) with negative rRT-PCR for SARS-CoV-2 RNA, 13 were male and 19 female (Table 1).

### 3.2. Primary Endpoint Results

Liposomal bLf-treated COVID-19 patients revealed a mean time to rRT-PCR SARS-CoV-2 RNA negative conversion of naso-oropharyngeal swab of 14.25 ± 6.0 days, shorter than that observed in the SOC-treated and the untreated with bLf COVID-19 groups (*p*-value < 0.0001) (Table 2).

SOC-treated COVID-19 patients showed a mean rRT-PCR SARS-CoV-2 RNA negative conversion of 27.13 ± 14.4 days, whereas untreated COVID-19 patients revealed a mean time to negative conversion of 32.61 ± 12.2 days (Table 2).

### 3.3. Secondary Endpoints Results

In 32 liposomal bLf-treated COVID-19 patients, we evaluated clinical symptoms before (T0) and after 30 days of bLf therapy (T2) (Table 3).

At T0, among 32 patients, 10 were asymptomatic and 22 were mild-to-moderate symptomatic patients. The most frequent symptoms were fatigue (43.75%), followed by arthralgia (37.5%), myalgia (37.5%), and cough (28.13%). The remaining symptoms were less frequent. At T0, 5 patients (15.63%) presented ageusia, and 5 patients (15.63%) presented anosmia. At T1, after 15 days of bLf supplement, 7 patients previously symptomatic became asymptomatic, with a total of 17 asymptomatic and 15 mild-to-moderate symptomatic patients. At T2, 6 other patients, previously symptomatic at T1, became asymptomatic, with a total of 23 asymptomatic and 9 mild-to-moderate symptomatic patients. The most frequent remaining symptom was fatigue, followed by rare manifestations related to diarrhea and skin manifestations. All patients presenting anosmia and ageusia at T0 showed a complete remission of the symptoms at T2 (Table 3).

Regarding safety assessment in the liposomal bLf-treated group, 2 patients (6.2%) showed gastrointestinal complaints related to liposomal bLf. The patients did not suspend liposomal bLf, and the adverse event resolved itself spontaneously.

In SOC-treated COVID-19 patients, we evaluated clinical symptoms before (T0) and after therapy (T2) (Table 4). At T0, the most frequent symptoms were cough (71.86%), followed by myalgia (62.5%) and arthralgia (59.38%). In particular, all of the patients with ageusia (11) and anosmia (12) at T0 did not achieve remission at T2 (Table 4).

### 3.4. Tertiary Endpoints Results

Among the evaluated parameters in the three COVID-19 patient groups and in healthy volunteers, no variation was observed except in the liposomal bLf- and SOC-treated groups. In particular, in the liposomal bLf-treated group, we noticed an improvement in the platelet count (T0: 239.63 ± 83.05; T2: 243.70 ± 65.5; ΔT2-T0: 10.05 ± 10.26) and a decrease in ALT (T0: 29.36 ± 22.7; T2: 23.52 ± 12.34; ΔT2-T0: −7.32 ± 4.36) and AST (T0: 24.36 ± 9.80; T2: 22.64 ± 8.33; ΔT2-T0: −2.68 ± 2.52), even if at a not significant level. Conversely, D-dimers showed a significant decrease between T2 and T0 (ΔT2-T0: −392.56 ± 142.71, *p*-value = 0.01) as well as ferritin (ΔT2-T0: −90.63 ± 48.49, *p*-value = 0.04) and serum IL-6 (ΔT2-T0: −2.52 ± 1.46, *p*-value = 0.05) (Table 5). IL-10 levels increased between T0 (8.67 ± 3.26) and T2 (11.42 ± 6.05) with no statistical significance (ΔT2-T0: 2.55 ± 2.09). TNF-α decreased between T2 (25.97 ± 21.74) and T0 (37.34 ± 19.95) with no statistical significance (ΔT2-T0: −12.92 ± 8.81).

Concerning SOC-treated patients, statistically significant variations were observed only in hemoglobin (ΔT2-T0: −2.22 ± 0.95, *p*-value = 0.05), red blood cells (ΔT2-T0: −0.43 ± 0.17, *p*-value = 0.05), and platelet count (ΔT2-T0: 89.56 ± 18.9, *p*-value = 0.001) (Table 6).

## 4. Discussion

In addition to vaccines, there is a significant need for therapies efficacious in the early phase or mild infection, since instantaneous benefits of such strategies could lead to the improvement of patient outcomes and prevention of hospital recovery [51]. Longer term benefits may involve prevention of the chronic consequences of infection as well as control of transmission by shortening the period of infectiousness.

Thus, we focused our attention on bLf, the antiviral activity of this natural glycoprotein in vitro being well-known [36,37,52,53] and recently demonstrated also against SARS-CoV-2 [7,34,35]. In this preliminary study, we orally administered 1 g/day of liposomal bLf as a potential agent in the management of asymptomatic and mild-to-moderate COVID-19 patients.

The ability of bLf to hinder viral infection is generally attributed to its binding to cell surface anionic components and/or viral particles. BLf is able to competitively bind to HSPGs, components of the host cell surface and identified as initial interaction sites for enveloped viruses such as SARS-CoV-2 [7,54,55], thus hindering viral adhesion and internalization [34]. Moreover, bLf can also bind directly to surface spike glycoproteins of SARS-CoV-2 particles, as demonstrated in silico [34].

In order to explore the helpful antiviral and immunomodulating effects of bLf and its possible role in the management of asymptomatic and mild-to-moderate COVID-19 patients, we designed a preliminary clinical trial to investigate the effect and safety of this natural glycoprotein belonging to innate immunity.

We focused our research on asymptomatic and mild-to-moderate COVID-19 patients, considering them a transmission reservoir with possible evolution to the most severe disease form [56]. Li et al. [57], analyzing the viral shedding dynamics in asymptomatic and mildly symptomatic patients infected with SARS-CoV-2, observed a long-term viral shedding, also in the convalescent phase of the disease, where specific antibody production to SARS-CoV-2 may not ensure viral clearance after hospital discharge. In their study, the median duration of viral shedding appeared shorter in pre-symptomatic patients (11.5 days) compared to asymptomatic (28 days) and mild symptomatic cases (31 days) [57]. Accordingly, we documented a significantly reduced mean time to rRT-PCR SARS-CoV-2 RNA negative conversion in a liposomal bLf-treated group compared to SOC-treated and untreated patients, suggesting a favoring of gradual viral clearance and clinical symptoms recovery with a potential decrease in the risk of transmission and contagion. This result is in agreement with the data obtained by Rosa et al. [58] in a survey based on real-life clinical practice conducted on ambulatory asymptomatic, paucisymptomatic, and moderate symptomatic COVID-19 patients treated with bLf, alone or as a supplementary agent. BLf oral administration, unloaded in liposomes, induces a time to SARS-CoV-2 RNA negativization similar to that observed with liposomal bLf (15 versus 14.25 days).

Although there are currently rare satisfactory markers for predicting worsening of the disease, some cytokines, including IL-6, IL-10, and TNF-α, and D-dimers levels have been described as biomarkers related to severe SARS-CoV-2 infection [59,60,61,62]. In our study, we identified suitable dysregulated blood parameters to consider as treatment target markers. Indeed, we found a statistically significant difference in liposomal bLf-treated COVID-19 patients regarding some blood parameters, including IL-6, D-dimers, and ferritin. Even if this result appears very interesting, it requires a randomized clinical trial on a larger number of patients to be confirmed.

Mainly, high serum IL-6 levels are considered to be associated with higher disease severity; IL-6 inhibitors, such as tocilizumab, have been used to treat severe COVID-19 patients [63,64]. The ability of bLf to down-regulate pro-inflammatory cytokines, such as IL-6, has already been established in both in vitro [65] and in vivo [66] models as well as in clinical trials [67]. To our knowledge, even though in a small sample size, this should be the first evidence showing an IL-6 down-regulation in COVID-19 patients after liposomal bLf treatment.

We also observed a statistically significant decline in D-dimers levels, crucial to define disease prognosis, possibly leading to a reduction in SARS-CoV-2 complications related to coagulation disturbance. Recently, it has been shown that hLf can regulate the activation of plasminogen and control the coagulation cascade with a remarkable antithrombotic activity [44]. This property could be relevant, considering that COVID-19 is a prothrombotic disease and that the severity of the coagulation parameters’ impairment is related to a poor prognosis. In light of this view, SARS-CoV-2 is able to activate a prominent prothrombotic state rarely observed in viral diseases. Patients affected by severe COVID-19 pneumonia are at a higher risk of imbalance of coagulation parameters and are, thus, treated with low molecular weight heparin or unfractionated heparin at doses registered for prevention of venous thromboembolism [45].

Our clinical experience could lead us to speculate a potential protective and safe role of an early treatment of liposomal bLf in COVID-19 patients to control the risk of a thromboembolic evolution of the disease. Lf can exert negative regulatory effects on cell migration through the inhibition of plasminogen activation and via regulation of fibrinolysis [44]. In addition, we observed an increased platelet count after liposomal bLf treatment. Indeed, COVID-19 induces thrombocytopenia, as SARS-CoV-2 seems to entrap megakaryocytes and block the release of platelets [68]. Liposomal bLf, by rebalancing platelet count, induces COVID-19 viral clearance.

Ferritin, besides reflecting levels of iron stores in healthy individuals, also performs an acute-phase protein up-regulated and elevated in both infectious and non-infectious inflammation. This has been reported to be relevant in COVID-19 for assessing disease severity and patient outcome [69,70]. In particular, serum ferritin concentration shows significantly higher values in COVID-19 patients with a worse outcome compared to those with a good outcome [71].

Iron chelators, such as Lf, have been repeatedly proposed as a potential therapeutic target during infections [72], and even in COVID-19 we assessed the reduction of ferritin levels during liposomal bLf administration, demonstrating its anti-inflammatory activity together with its iron chelating ability, which is pivotal for bacterial and viral replication and at the basis of its antibacterial and antiviral activity [23,31,36,37].

Liver function is known to be disturbed in COVID-19, and a meta-analysis showed that 16% and 20% of patients with COVID-19 had ALT and AST levels higher than the normal range [73]. Liver biochemistry abnormality in COVID-19 patients could be ascribed to several factors, such as direct hepatocyte injury by the virus, drug-induced liver injury, hypoxic-ischemic microcirculation disorder, and underlying liver diseases [62]. In our study, we observed that liposomal bLf reduced transaminase levels, even if at a not significant level, thus decreasing the risk of liver injury among COVID-19 patients, which is a frequent complication in severe forms of SARS-CoV-2 [74].

Regarding clinical symptoms recovery, in the liposomal bLf-treated COVID-19 patients we observed a gradual reduction of all symptoms, with the exception of fatigue, which persisted in 18.75% of patients (Table 3). We explained this result considering patient age and concomitant comorbidities, which could create a bias to identify COVID-19 symptoms. On the other hand, in SOC-treated patients, we observed a partial symptoms recovery and, particularly, the persistence of anosmia and ageusia in all affected patients, even at the end of treatment (Table 4).

Concerning liposomal bLf safety, we reported gastrointestinal complaints in two patients as occasional findings that did not lead to treatment discontinuation. Therefore, we concluded that bLf was safe and well tolerated among our study population.

Regarding the SOC regimen group we observed limited adverse events related to the administration of hydroxychloroquine, even though its use is controversial, as several meta-analyses pointed out its intrinsic safety risks, especially in vulnerable populations, and its inefficacy in mitigating COVID-19 symptoms. Indeed, hydroxychloroquine has been related to gastric symptoms, retinopathy, and cardiomyopathy [75,76]. As a matter of fact, in a paper by Mercuro et al. [48], patients who received hydroxychloroquine for the treatment of pneumonia associated with COVID-19 were at high risk of corrected QT (QTc) prolongation, and the combination with azithromycin was associated with greater changes in QTc [48]. Overall, the use of hydroxychloroquine alone or in combination with azithromycin was not effective in treating COVID-19 hospitalized patients. In addition, it was associated with higher mortality rates in comparison with the control group [76].

In our analysis, we used formulations containing bLf embedded in liposomes. The inclusion of bLf in preserving structures, such as liposomes, reduces gastric and intestinal enzymatic digestion while maintaining its integrity and, therefore, its biological functionality [77,78]. Indeed, the bLf at 5% of iron saturation form is best suited to obtain the maximum chelating effect. Liposomal bLf is intranasally and orally administered, because it exerts two main functions dependent on topical or oral systemic administration. The topical intranasal administration (about 16 mg/nostril/day) is related to bLf binding with HSPGs of host cells and spike glycoproteins. This binding establishes a protective barrier against viral infection. Conversely, oral systemic administration of bLf (1 g/day) is related to the anti-inflammatory and anti-thrombotic activities. Of note, the anti-inflammatory activity also decreases intracellular iron overload, which, in turn, facilitates viral multiplication.

One of the limitations of our study was the small sample size of COVID-19 patients. Further studies on larger samples are needed to better evaluate the role of bLf in treating SARS-CoV-2 and to define the best treatment: bLf alone or as a supplementary agent.

Considering the risk of COVID-19 relapse [79], we also suggest additional long-term studies to evaluate the maintenance of viral clearance via continuous administration of bLf.

Only after the randomized clinical trials to confirm its efficacy could bLf be considered as an effective treatment in asymptomatic and mild-to-moderate COVID-19 patients. This could not only improve patient outcomes and prevention of hospital recovery, but also hinder chronic consequences of infection and disease transmission, especially by shortening the period of infectiousness.

## Figures and Tables

**Table 1:** Demographic data of the patients enrolled in the preliminary clinical trial. BLf = bovine lactoferrin; SOC = standard of care; SD = standard deviation.

Demographic Data		Liposomal bLf-Treated COVID-19 Group		SOC-Treated COVID-19 Group		Untreated COVID-19 Group		Control Group (COVID-19 Negative)	
		Mean ± SD	N (%)	Mean ± SD	N (%)	Mean ± SD	N (%)	Mean ± SD	N (%)
Age		54.56 ± 16.86		49.9 ± 13.20		41.32 ± 11.77		52.80 ± 15.54	
Sex	Male		14 (44%)		17 (53%)		10 (36%)		13 (41%)
	Female		18 (56%)		15 (47%)		18 (64%)		19 (59%)
Asymptomatic patients			10 (31%)		3 (10%)		12 (43%)		
Mild-to-moderate patients			22 (68.7%)		29 (90%)		16 (57%)		

**Table 2:** Mean time and standard deviation (SD) to reverse transcriptase real time (rRT)-PCR SARS-CoV-2 RNA negative conversion in liposomal bovine lactoferrin (bLf)-treated, standard of care (SOC)-treated, or untreated COVID-19 patients.

	Number of Enrolled Patients	Mean Time ± SD to rRT-PCR SARS-CoV-2 RNA Negative Conversion of Naso-Oropharyngeal Swab	*p*-Value
Liposomal bLf-treated COVID-19 patients	32	14.25 ± 6.0	
SOC-treated COVID-19 patients	32	27.13 ± 14.4	<0.0001
Untreated COVID-19 patients	28	32.61 ± 12.2	<0.0001

**Table 3:** Symptoms in liposomal bovine lactoferrin (bLf)-treated COVID-19 group at T0 (baseline) and at T2 (end of the study).

	Cough	Rhinorrhea	Ageusia	Anosmia	Diarrhea	Myalgia	Arthralgia	Fatigue	Headache	Vomit	Conjunctivitis	Skin Manifestation
T0	9 (28.13%)	2 (6.25%)	5 (15.63%)	5 (15.63%)	3 (9.36%)	12 (37.5%)	12 (37.5%)	14 (43.75%)	3 (9.36%)	0	3 (9.36%)	3 (9.36%)
T2	0	0	0	0	1 (3.0%)	0	0	6 (18.75%)	0	0	0	1 (3.0%)

**Table 4:** Symptoms in standard of care (SOC)-treated COVID-19 group at T0 (baseline) and at T2 (end of the study).

	Cough	Rhinorrhea	Agenusia	Anosmia	Diarrhea	Myalgia	Arthralgia	Fatigue	Headache	Vomit	Conjunctivitis	Skin Manifestation
T0	23 (71.86%)	4 (12.5%)	11 (34.38%)	11 (34.38%)	3 (9.38%)	20 (62.5%)	19 (59.38%)	14 (43.75%)	3 (9.38%)	0	4 (12.5%)	2 (6.25%)
T2	5 (15.63%)	0	11 (34.38%)	11 (34.38%)	1 (3.13%)	7 (21.88%)	6 (18.75%)	6 (18.75%)	4 (12.5%)	0	2 (6.25%)	0

**Table 5:** Parameters showing significant variations between T2 and T0 in liposomal bovine lactoferrin (bLf)-treated COVID-19 patients.

Statistical Analysis	D-Dimers (ng/mL) Δ(T2-T0)	Ferritin (ng/mL) Δ(T2-T0)	IL-6 (pg/mL) Δ(T2-T0)
Mean	−392.56	−90.63	−2.52
Variance	366600.7	42319.88	36.31
Standard Deviation	142.71	48.49	1.46
t-Student	−2.75	−1.87	−1.73
*p*-value	0.01	0.04	0.05

**Table 6:** Parameters showing significant variations between T2 and T0 in standard of care (SOC)-treated COVID-19 patients.

Statistical Analysis	Hemoglobin (g/dL) Δ(T2-T0)	Red Blood Cell Count (10^6^/µL) Δ(T2-T0)	Platelet Count (10^3^/µL) Δ(T2-T0)
Mean	−2.22	−0.43	89.56
Variance	14.57	0.46	5713.33
Standard Deviation	0.95	0.17	18.9
t-Student	−2.337	−2.529	4.739
*p*-value	0.05	0.05	0.001

## Data Availability

The data presented in this study are available in the article.

## References

[1] Lu R., Zhao X., Li J., Niu P., Yang B., Wu H., Wang W., Song H., Huang B., Zhu N. (2020). Genomic characterisation and epidemiology of 2019 novel coronavirus: Implications for virus origins and receptor binding. Lancet.

[2] Tian X., Li C., Huang A., Xia S., Lu S., Shi Z., Lu L., Jiang S., Yang Z., Wu Y. (2020). Potent binding of 2019 novel coronavirus spike protein by a SARS coronavirus-specific human monoclonal antibody. Emerg. Microbes Infect..

[3] Lan J., Ge J., Yu J., Shan S., Zhou H., Fan S., Zhang Q., Shi X., Wang Q., Zhang L. (2020). Structure of the SARS-CoV-2 spike receptor-binding domain bound to the ACE2 receptor. Nature.

[4] Cui J., Li F., Shi Z.-L. (2019). Origin and evolution of pathogenic coronaviruses. Nat. Rev. Microbiol..

[5] Li F. (2016). Structure, Function, and Evolution of Coronavirus Spike Proteins. Annu. Rev. Virol..

[6] Lang J., Yang N., Deng J., Liu K., Yang P., Zhang G., Jiang C. (2011). Inhibition of SARS pseudovirus cell entry by lactoferrin binding to heparan sulfate proteoglycans. PLoS ONE.

[7] Hu Y., Meng X., Zhang F., Xiang Y., Wang J. (2021). The In Vitro antiviral activity of lactoferrin against common human coronaviruses and SARS-CoV-2 is mediated by targeting the heparan sulfate co-receptor. Emerg. Microbes Infect..

[8] Butowt R., Bilinska K. (2020). SARS-CoV-2: Olfaction, Brain Infection, and the Urgent Need for Clinical Samples Allowing Earlier Virus Detection. ACS Chem. Neurosci..

[9] Xu H., Zhong L., Deng J., Peng J., Dan H., Zeng X., Li T., Chen Q. (2020). High expression of ACE2 receptor of 2019-nCoV on the epithelial cells of oral mucosa. Int. J. Oral Sci..

[10] Zou X., Chen K., Zou J., Han P., Hao J., Han Z. (2020). Single-cell RNA-seq data analysis on the receptor ACE2 expression reveals the potential risk of different human organs vulnerable to 2019-nCoV infection. Front. Med..

[11] Loganathan S., Kuppusamy M., Wankhar W., Gurugubelli K.R., Mahadevappa V.H., Lepcha L., Choudary A.K. (2021). Angiotensin-converting enzyme 2 (ACE2): COVID 19 gate way to multiple organ failure syndromes. Respir. Physiol. Neurobiol..

[12] Chen N., Zhou M., Dong X., Qu J., Gong F., Han Y., Li T., Chen Q. (2020). Epidemiological and clinical characteristics of 99 cases of 2019 novel coronavirus pneumonia in Wuhan, China: A descriptive study. Lancet.

[13] Huang B., Ling R., Cheng Y., Wen J., Dai Y., Huang W., Zhang S., Lu X., Luo Y., Jiang Y.Z. (2020). Characteristics of the coronavirus disease 2019 and related therapeutic options. Mol. Ther. Methods Clin. Dev..

[14] Chan J.F., Yuan S., Kok K.H., To K.K., Chu H., Yang J., Xing F., Liu J., Yip C.C., Poon R.W. (2020). A familial cluster of pneumonia associated with the 2019 novel coronavirus indicating person-to-person transmission: A study of a family cluster. Lancet.

[15] Chen C., Zhang X.R., Ju Z.Y., He W.F. (2020). [Advances in the research of mechanism and related immunotherapy on the cytokine storm induced by coronavirus disease 2019]. Zhonghua Shao Shang Za Zhi.

[16] Guan W.J., Ni Z.Y., Hu Y., Liang W.H., Ou C.Q., He J.X., Liu L., Shan H., Lei C.L., Hui D. (2020). Clinical characteristics of coronavirus disease 2019 in China. N. Engl. J. Med..

[17] Tsai P.H., Lai W.Y., Lin Y.Y., Luo Y.H., Lin Y.T., Chen H.K., Chen Y.M., Lai Y.C., Kuo L.C., Chen S.D. (2021). Clinical manifestation and disease progression in COVID-19 infection. J. Chin. Med. Assoc..

[18] Li L.-Q., Huang T., Wang Y.-Q., Wang Z.-P., Liang Y., Huang T.-B., Zhang H.Y., Sun W., Wang Y. (2020). COVID-19 patients’ clinical characteristics, discharge rate, and fatality rate of meta-analysis. J. Med. Virol..

[19] Merad J., Martin J.C. (2020). Pathological inflammation in patients with COVID-19: A key role for monocytes and macrophages. Nat. Rev. Immunol..

[20] Goldberg M.F., Goldberg M.F., Cerejo R., Tayal A.H. (2020). Cerebrovascular Disease in COVID-19. AJNR Am. J. Neuroradiol..

[21] Liu W., Li H. (2020). COVID-19: Attacks the 1-beta chain of hemoglobin and captures the porphyrin to inhibit human heme metabolism. ChemRxiv.

[22] Zhou F., Yu T., Du R., Fan G., Liu Y., Liu Z., Xiang J., Wang Y., Song B., Gu X. (2020). Clinical course and risk factors for mortality of adult inpatients with COVID-19 in Wuhan, China: A retrospective cohort study. Lancet.

[23] Mancinelli R., Rosa L., Cutone A., Lepanto M.S., Franchitto A., Onori P., Gaudio E., Valenti P. (2020). Viral Hepatitis and Iron Dysregulation: Molecular Pathways and the Role of Lactoferrin. Molecules.

[24] COVID-19 Treatment Guidelines Panel Coronavirus Disease 2019 (COVID-19) Treatment Guidelines. National Institutes of Health. https://www.covid19treatmentguidelines.nih.gov/..

[25] RECOVERY Collaborative Group (2020). Lopinavir-ritonavir in patients admitted to hospital with COVID-19 (RECOVERY): A randomised, controlled, open-label, platform trial. Lancet.

[26] Tahir Ul Qamar M., Alqahtani S.M., Alamri M.A., Chen L.L. (2020). Structural basis of SARS-CoV-2 3CLpro and antiCOVID-19 drug discovery from medicinal plants. J. Pharm. Anal..

[27] Liu X., Wang X.J. (2020). Potential inhibitors against 2019-nCoV coronavirus M protease from clinically approved medicines. J. Genet. Genom..

[28] Wang M., Cao R., Zhang L., Yang X., Liu J., Xu M., Shi Z., Hu Z., Zhong W., Xiao G. (2020). Remdesivir and chloroquine effectively inhibit the recently emerged novel coronavirus (2019-nCoV) In Vitro. Cell Res..

[29] Vincent M.J., Bergeron E., Benjannet S., Erickson B.R., Rollin P.E., Ksiazek T.G., Seidah N.G., Nichol S.T. (2005). Chloroquine is a potent inhibitor of SARS coronavirus infection and spread. Virol. J..

[30] Rosa L., Cutone A., Lepanto M.S., Paesano R., Valenti P. (2017). Lactoferrin: A Natural Glycoprotein Involved in Iron and Inflammatory Homeostasis. Int. J. Mol. Sci..

[31] Valenti P., Antonini G. (2005). Lactoferrin: An important host defence against microbial and viral attack. Cell. Mol. Life Sci..

[32] U.S. FDA GRN 000465 [Cow’s Milk-Derived Lactoferrin, Tokyo, Japan: Morinaga Milk Industry Co., Ltd.]. Silver Spring, MD, USA. Food and Drug Administration (U.S. FDA), Center for Food Safety & Applied Nutrition (CFSAN), Office of Food Additive Safety. 2014. http://www.accessdata.fda.gov/scripts/fdcc/index.cfm?set=GRASNotices&id=465.

[33] EFSA Panel on Dietetic Products, Nutrition and Allergies (NDA) (2012). Scientific Opinion on bovine lactoferrin. EFSA J..

[34] Campione E., Lanna C., Cosio T., Rosa L., Conte M.P., Iacovelli F., Romeo A., Falconi M., Del Vecchio C., Franchin E. (2021). Lactoferrin Against SARS-CoV-2: In vitro and in silico Evidences. Front. Pharmacol..

[35] Salaris C., Scarpa M., Elli M., Bertolini A., Guglielmetti S., Pregliasco F., Blandizzi C., Brun P., Castagliuolo I. (2021). Protective Effects of Lactoferrin against SARS-CoV-2 Infection In Vitro. Nutrients.

[36] Berlutti F., Pantanella F., Natalizi T., Frioni A., Paesano R., Polimeni A., Valenti P. (2011). Antiviral properties of lactoferrin—A natural immunity molecule. Molecules.

[37] Wakabayashi H., Oda H., Yamauchi K., Abe F. (2014). Lactoferrin for prevention of common viral infections. J. Infect. Chemother..

[38] Ashida K., Sasaki H., Suzuki Y.A., Lönnerdal B. (2004). Cellular internalization of lactoferrin in intestinal epithelial cells. Biometals.

[39] Lepanto M.S., Rosa L., Paesano R., Valenti P., Cutone A. (2019). Lactoferrin in Aseptic and Septic Inflammation. Molecules.

[40] Suzuki Y.A., Wong H., Ashida K.-Y., Schryvers A.B., Lönnerdal B. (2008). The N1 domain of human lactoferrin is required for internalization by caco-2 cells and targeting to the nucleus. Biochemistry.

[41] Liao Y., Jiang R., Lönnerdal B. (2012). Biochemical and molecular impacts of lactoferrin on small intestinal growth and development during early life. Biochem. Cell Biol..

[42] Kruzel M.L., Zimecki M., Actor J.K. (2017). Lactoferrin in a Context of Inflammation-Induced Pathology. Front. Immunol..

[43] Campione E., Cosio T., Rosa L., Lanna C., Di Girolamo S., Gaziano R., Valenti P., Bianchi L. (2020). Lactoferrin as Protective Natural Barrier of Respiratory and Intestinal Mucosa against Coronavirus Infection and Inflammation. Int. J. Mol. Sci..

[44] Zwirzitz A., Reiter M., Skrabana R., Ohradanova-Repic A., Majdic O., Gutekova M., Cehlar O., Petrovčíková E., Kutejova E., Stanek G. (2018). Lactoferrin is a natural inhibitor of plasminogen activation. J. Biol. Chem..

[45] Marietta M., Coluccio V., Luppi M. (2020). COVID-19, coagulopathy and venous thromboembolism: More questions than answers. Intern. Emerg. Med..

[46] Xu Y.-H., Dong J.-H., An W.-M., Lv X.-Y., Yin X.-P., Zhang J.-Z., Dong L., Ma X., Zhang H.J., Gao B.L. (2020). Clinical and computed tomographic imaging features of novel coronavirus pneumonia caused by SARS-CoV-2. J. Infect..

[47] Nguyen L.S., Dolladille C., Drici M.D., Fenioux C., Alexandre J., Mira J.P., Moslehi J.J., Roden D.M., Funck-Brentano C., Salem J.E. (2020). Cardiovascular toxicities associated with hydroxychloroquine and azithromycin: An analysis of the World Health Organization pharmacovigilance database. Circulation.

[48] Mercuro N.J., Yen C.F., Shim D.J., Maher T.R., McCoy C.M., Zimetbaum P.J., Gold H.S. (2020). Risk of QT interval prolongation associated with use of hydroxychloroquine with or without concomitant azithromycin among hospitalized patients testing positive for coronavirus disease 2019 (COVID-19). JAMA Cardiol..

[49] Chorin E., Wadhwani L., Magnani S., Dai M., Shulman E., Nadeau-Routhier C., Knotts R., Bar-Cohen R., Kogan E., Barbhaiya C. (2020). QT interval prolongation and torsade de pointes in patients with COVID-19 treated with hydroxychloroquine/azithromycin. Heart Rhythm..

[50] Rosa L., Lepanto M.S., Cutone A., Siciliano R.A., Paesano R., Costi R., Musci G., Valenti P. (2020). Influence of oral administration mode on the efficacy of commercial bovine Lactoferrin against iron and inflammatory homeostasis disorders. Biometals.

[51] Kim P.S., Read S.W., Fauci A.S. (2020). Therapy for Early COVID-19: A Critical Need. JAMA.

[52] van der Strate B.W., Beljaars L., Molema G., Harmsen M.C., Meijer D.K. (2001). Antiviral activities of lactoferrin. Antiviral Res..

[53] Ng T.B., Cheung R.C.F., Wong J.H., Wang Y., Ip D.T.M., Wan D.C.C., Xia J. (2015). Antiviral activities of whey proteins. Appl. Microbiol. Biotechnol..

[54] Liu L., Chopra P., Li X., Wolfert M.A., Tompkins S.M., Boons G.J. (2020). SARS-CoV-2 spike protein binds heparan sulfate in a length and sequence dependent manner. BiorXiv.

[55] Zhang Q., Chen C.Z., Swaroop M., Xu M., Wang L., Lee J., Wang A.Q., Pradhan M., Hagen N., Chen L. (2020). Heparan sulfate assists SARS-CoV-2 in cell entry and can be targeted by approved drugs In Vitro. Cell. Discov..

[56] Jiang X.-L., Zhang X.-L., Zhao X.-N., Li C.-B., Lei J., Kou Z.-Q., Sun W.K., Hang Y., Gao F., Ji S.X. (2020). Transmission Potential of Asymptomatic and Paucisymptomatic Severe Acute Respiratory Syndrome Coronavirus 2 Infections: A 3-Family Cluster Study in China. J. Infect. Dis..

[57] Li W., Su Y.-Y., Zhi S.-S., Huang J., Zhuang C.-L., Bai W.-Z., Wan Y., Meng X.R., Zhang L., Zou Y.B. (2020). Viral shedding dynamics in asymptomatic and mildly symptomatic patients infected with SARS-CoV-2. Clin. Microbiol. Infect..

[58] Rosa L., Tripepi G., Naldi E., Aimati M., Santangeli S., Venditto F., Caldarelli M., Valenti P. (2021). Ambulatory COVID-19 Patients Treated with Lactoferrin as a Supplementary Antiviral Agent: A Preliminary Study. J. Clin. Med..

[59] Aziz M., Fatima R., Assaly R. (2020). Elevated interleukin-6 and severe COVID-19: A meta-analysis. J. Med. Virol..

[60] Li X., Geng M., Peng Y., Meng L., Lu S. (2020). Molecular immune pathogenesis and diagnosis of COVID-19. J. Pharm. Anal..

[61] Tang N., Li D., Wang X., Sun Z. (2020). Abnormal coagulation parameters are associated with poor prognosis in patients with novel coronavirus pneumonia. J. Thromb. Haemost..

[62] Xu Z., Shi L., Wang Y., Zhang J., Huang L., Zhang C., Liu S., Zhao P., Liu H., Zhu L. (2020). Pathological findings of COVID-19 associated with acute respiratory distress syndrome. Lancet Respir. Med..

[63] Cortegiani A., Ippolito M., Greco M., Granone V., Protti A., Gregoretti C., Giarratano A., Einav S., Cecconi M. (2020). Rationale and evidence on the use of tocilizumab in COVID-19: A systematic review. Pulmonology.

[64] Maeda T., Obata R., Do D.R., Kuno T. (2021). The Association of Interleukin-6 value, Interleukin inhibitors and Outcomes of Patients with COVID-19 in New York City. J. Med. Virol..

[65] Cutone A., Rosa L., Lepanto M.S., Scotti M.J., Berlutti F., Bonaccorsi di Patti M.C., Musci G., Valenti P. (2017). Lactoferrin Efficiently Counteracts the Inflammation-Induced Changes of the Iron Homeostasis System in Macrophages. Front. Immunol..

[66] Valenti P., Frioni A., Rossi A., Ranucci S., De Fino I., Cutone A., Rosa L., Bragonzi A., Berlutti F. (2017). Aerosolized bovine lactoferrin reduces neutrophils and pro-inflammatory cytokines in mouse models of *Pseudomonas aeruginosa* lung infections. Biochem. Cell Biol..

[67] Lepanto M.S., Rosa L., Cutone A., Conte M.P., Paesano R., Valenti P. (2018). Efficacy of Lactoferrin Oral Administration in the Treatment of Anemia and Anemia of Inflammation in Pregnant and Non-pregnant Women: An Interventional Study. Front. Immunol..

[68] Thachil J. (2020). What do monitoring platelet counts in COVID-19 teach us?. J. Thromb. Haemost..

[69] Bolondi G., Russo E., Gamberini E., Circelli A., Meca M.C.C., Brogi E., Viola L., Bissoni L., Poletti V., Agnoletti V. (2020). Iron metabolism and lymphocyte characterisation during Covid-19 infection in ICU patients: An observational cohort study. World J. Emerg. Surg..

[70] Kappert K., Jahić A., Tauber R. (2020). Assessment of serum ferritin as a biomarker in COVID-19: Bystander or participant? Insights by comparison with other infectious and non-infectious diseases. Biomarkers.

[71] Satış H., Özger H.S., Aysert Yıldız P., Hızel K., Gulbahar Ö., Erbaş G., Aygencel G., Guzel Tunccan O., Öztürk M.A., Dizbay M. (2021). Prognostic value of interleukin-18 and its association with other inflammatory markers and disease severity in COVID-19. Cytokine.

[72] Dalamaga M., Karampela I., Mantzoros C.S. (2020). Commentary: Could iron chelators prove to be useful as an adjunct to COVID-19 Treatment Regimens?. Metab. Clin. Exp..

[73] Deng X., Liu B., Li J., Zhang J., Zhao Y., Xu K. (2020). Blood biochemical characteristics of patients with coronavirus disease 2019 (COVID-19): A systemic review and meta-analysis. Clin. Chem. Lab. Med..

[74] Wang Q., Zhao H., Liu L.-G., Wang Y.-B., Zhang T., Li M.-H., Xu Y.L., Gao G.J., Xiong H.F., Fan Y. (2020). Pattern of liver injury in adult patients with COVID-19: A retrospective analysis of 105 patients. Mil. Med. Res..

[75] Kim M.S., An M.H., Kim W.J., Hwang T.H. (2020). Comparative efficacy and safety of pharmacological interventions for the treatment of COVID-19: A systematic review and network meta-analysis. PLoS Med..

[76] Amani B., Khanijahani A., Amani B. (2021). Hydroxychloroquine plus standard of care compared with standard of care alone in COVID-19: A meta-analysis of randomized controlled trials. Sci. Rep..

[77] Liu W., Ye A., Liu W., Liu C., Singh H. (2013). Stability during In Vitro digestion of lactoferrin-loaded liposomes prepared from milk fat globule membrane-derived phospholipids. J. Dairy Sci..

[78] Zhang J., Han J., Ye A., Liu W., Tian M., Lu Y., Wu K., Liu J., Lou M.P. (2019). Influence of Phospholipids Structure on the Physicochemical Properties and In Vitro Digestibility of Lactoferrin-Loaded Liposomes. Food Biophys..

[79] Prévost J., Gasser R., Beaudoin-Bussières G., Richard J., Duerr R., Laumaea A., Anand S.P., Goyette G., Benlarbi M., Ding S. (2020). Cross-sectional evaluation of humoral responses against SARS-CoV-2 Spike. Cell. Rep. Med..

